# Metropolitan Hospital Sunday Fund

**Published:** 1906-06-23

**Authors:** 


### METROPOLITAN HOSPITAL SUNDAY FUND.

The contributions received at the Mansion House towards the Hospital Sunday Fund amount to £12,000. Amongst the sums received are the following: St. Michael, Chester Square, per Canon Fleming, £1,353; St. Paul's Cathedral, £439; William Herring, Esq., £1,000; Messrs. Watts, Watts and Co., £210; W. D. Graham-Menzies, Esq., £200; A. G. P., £200; Delta, £200; Miss Arden, £100; F. G. D., £100; Messrs. J. S. Morgan and Co., £100; Alex. Miller, Esq., £100; Anon. £100; St. Mary, Shortlands, £101; St. Andrew's Presbyterian Church, Frognal, £84; SS. Anne and Mary, Wandsworth, £83; St. Augustine, Queen's Gate, £71; Holy Trinity, Eltham, £58; Christ Church, Newgate Street, £53; Messrs. Vavasseur and Co., £52 10s.; St. Andrew, Stoke Newington, £52; Mrs. Gooch, £50; G. P. Gooch, Esq., M.P., £50, per Rev. W. R. Finlay; Miss M. E. Druce, £50; F. Fleischmann, Esq., £50; the Earl of Cranbrook, £50; St. John, Sidcup, £44; St. Michael, Bletchworth, £42; St. Paul, Wimbledon Park, £42; St. Mary, Hayes, £37; St. Matthew, Westminster, £37; Christ Church, Wanstead, £35; Chiswick Parish Church, £35; St. Mary (Old Church), Stoke Newington, £35; St. Luke, Hornsey, £32; St. George, Bloomsbury, £31; C. W. Drabble, Esq., £30; Chislehurst Wesleyan Church, £29; Carmelite Church, Kensington, £27; Effra Road Unitarian Church, Brixton, £26; H. Dent Brocklehurst, Esq., £25; Miss de Wend, £25; St. James, Knatchbull Road, £24; Upper Holloway Baptist Church, £24; Finsbury Park Congregational Church, £24; Duke of York's School, £25; St. Giles, Camberwell, £21; Epsom Parish Church, £21. Holy Trinity, Dartford, £20.

